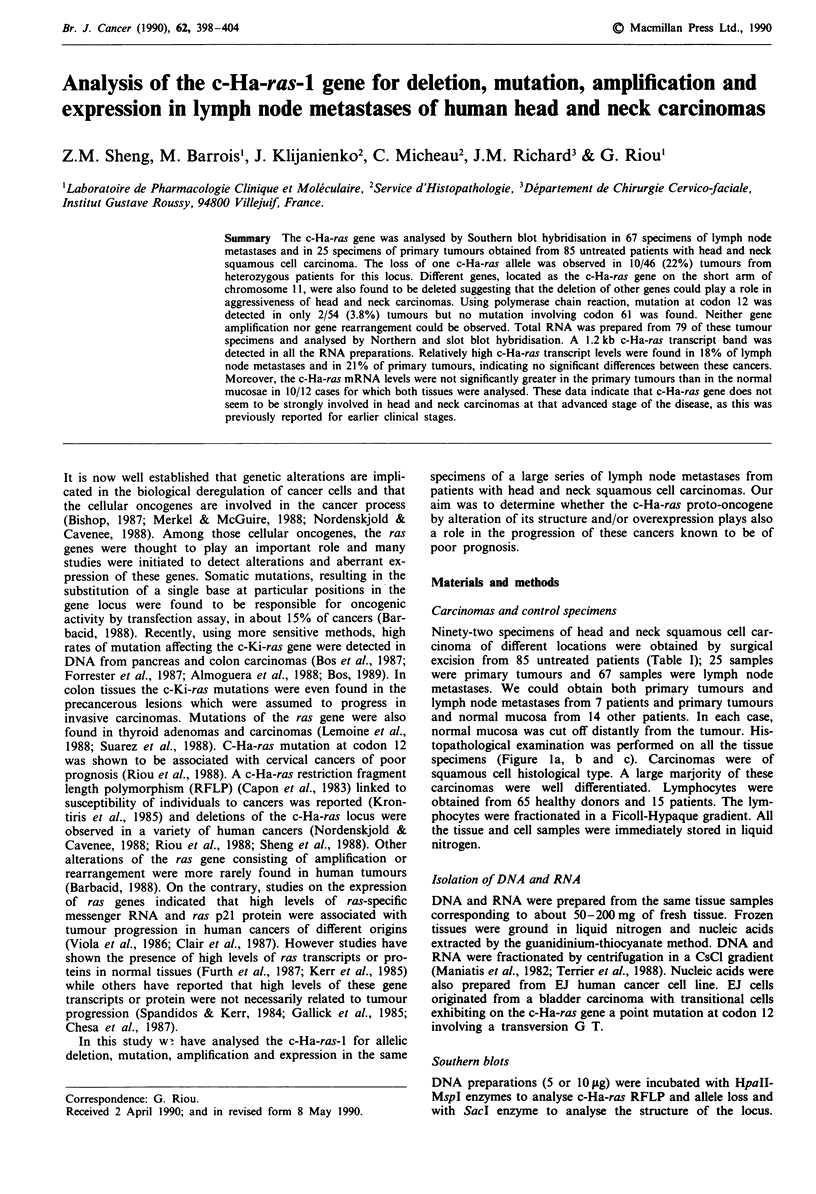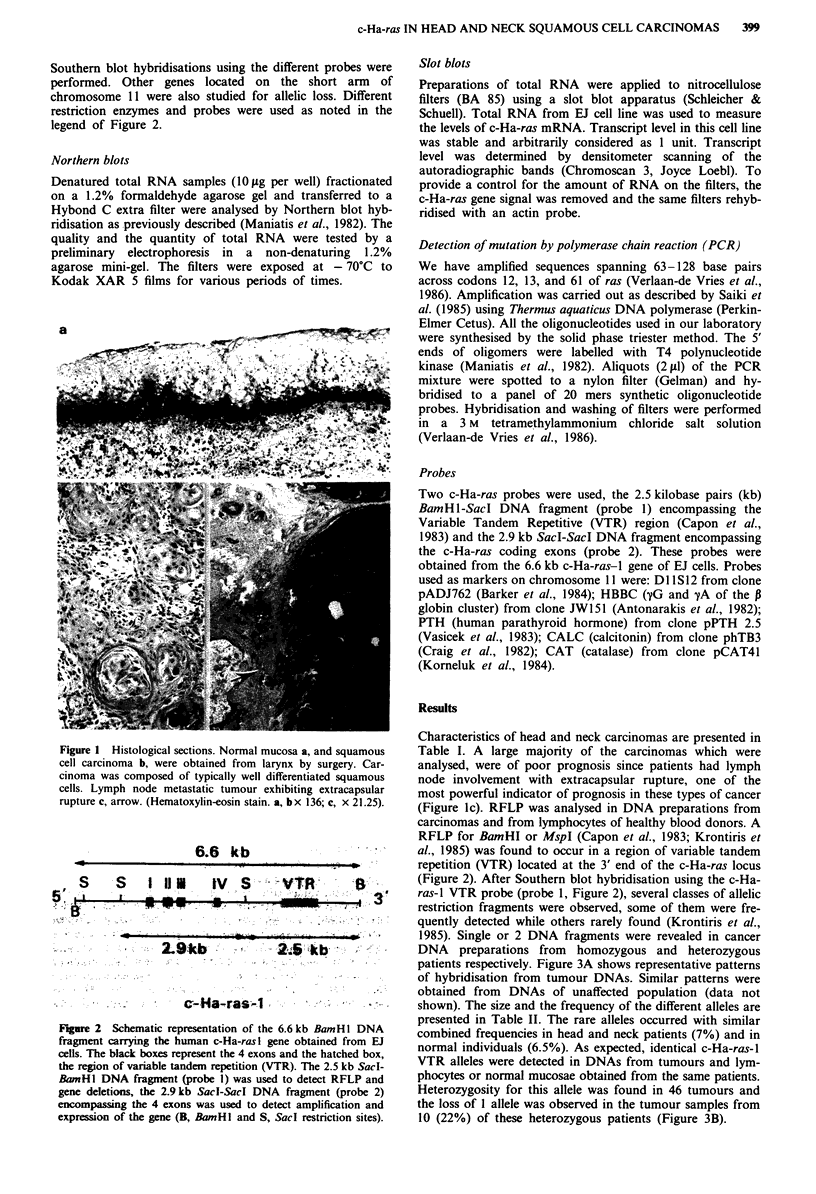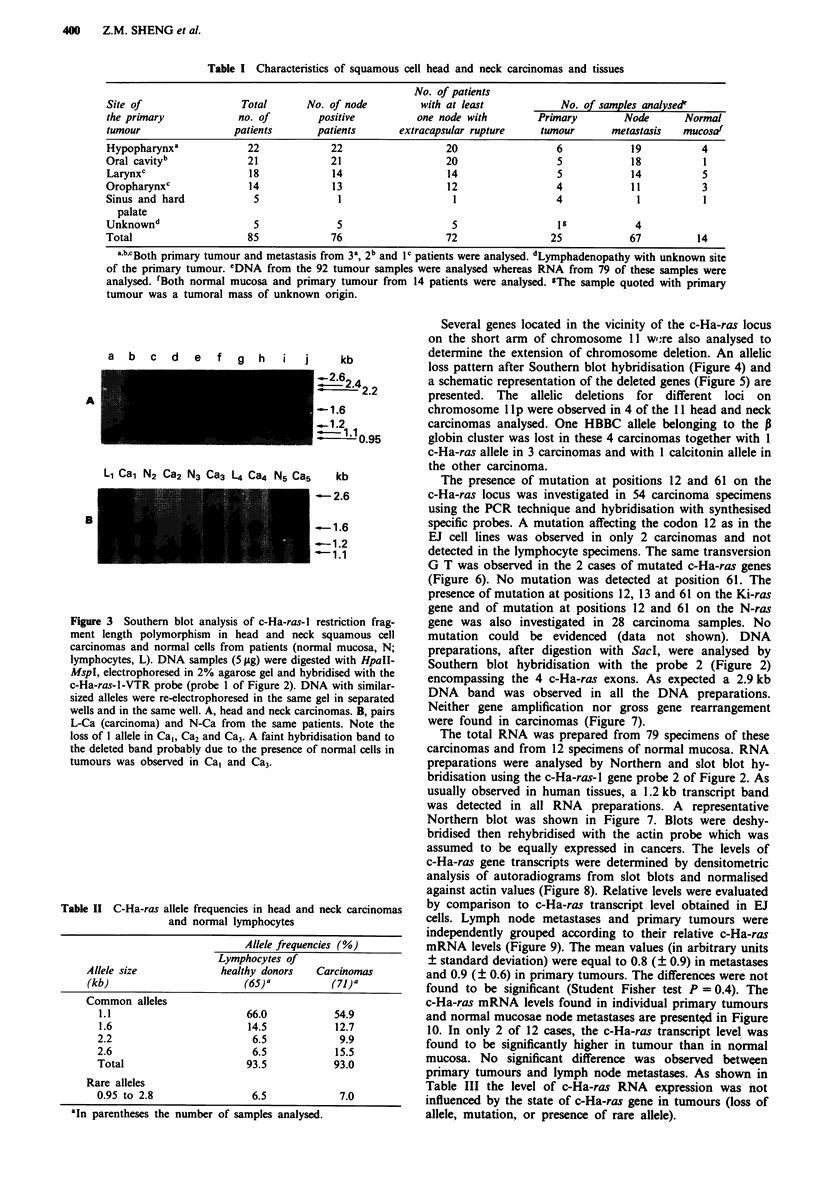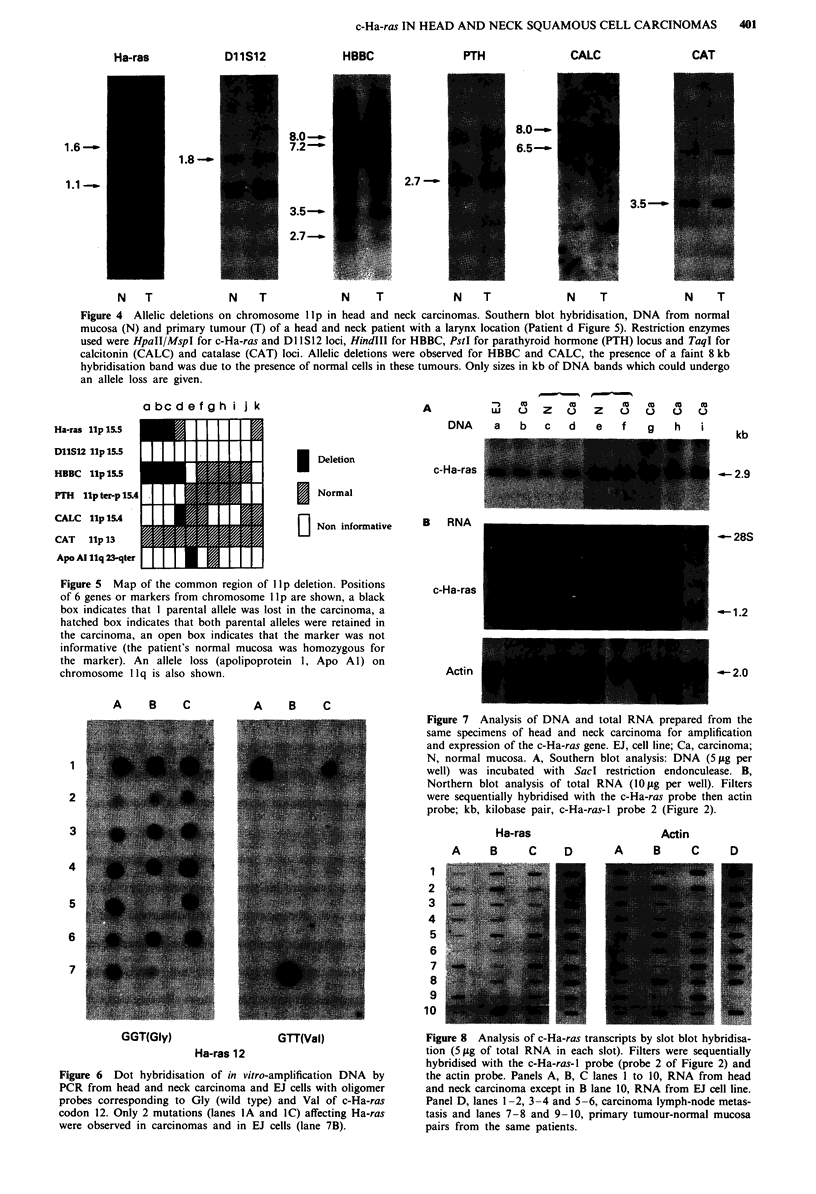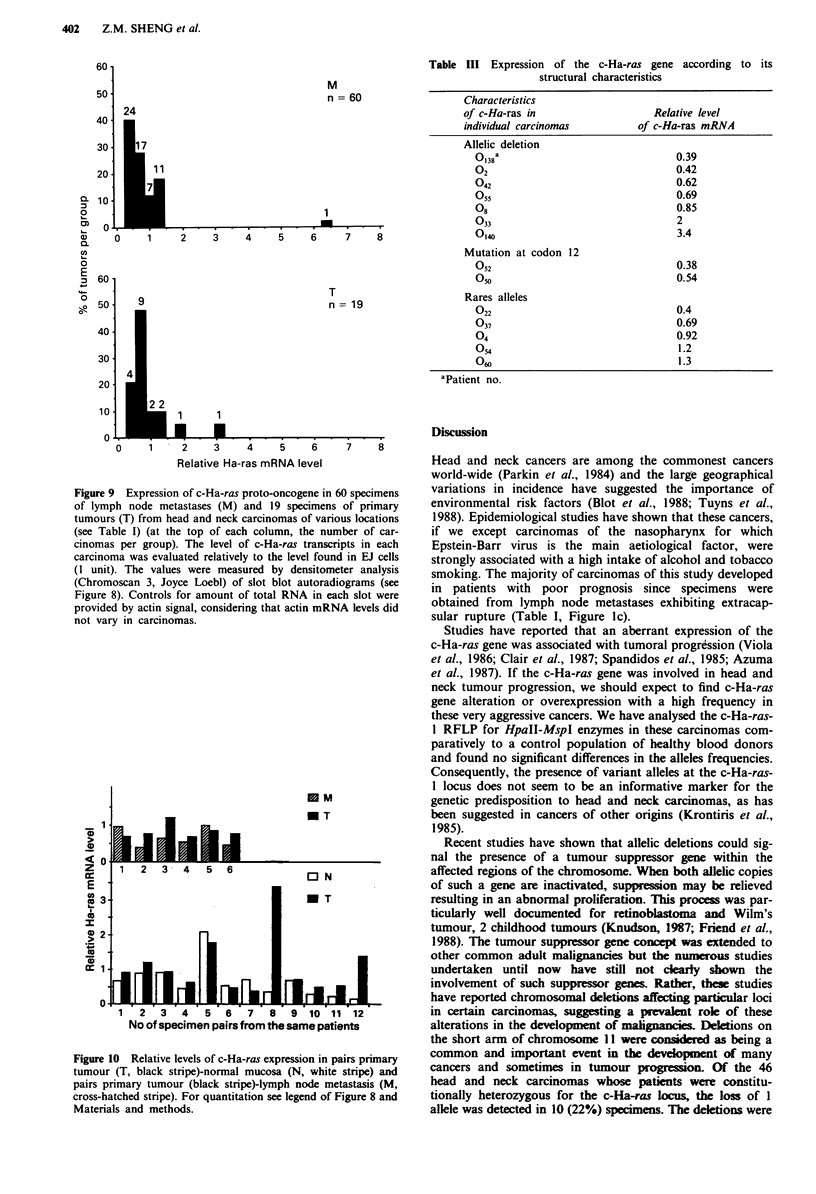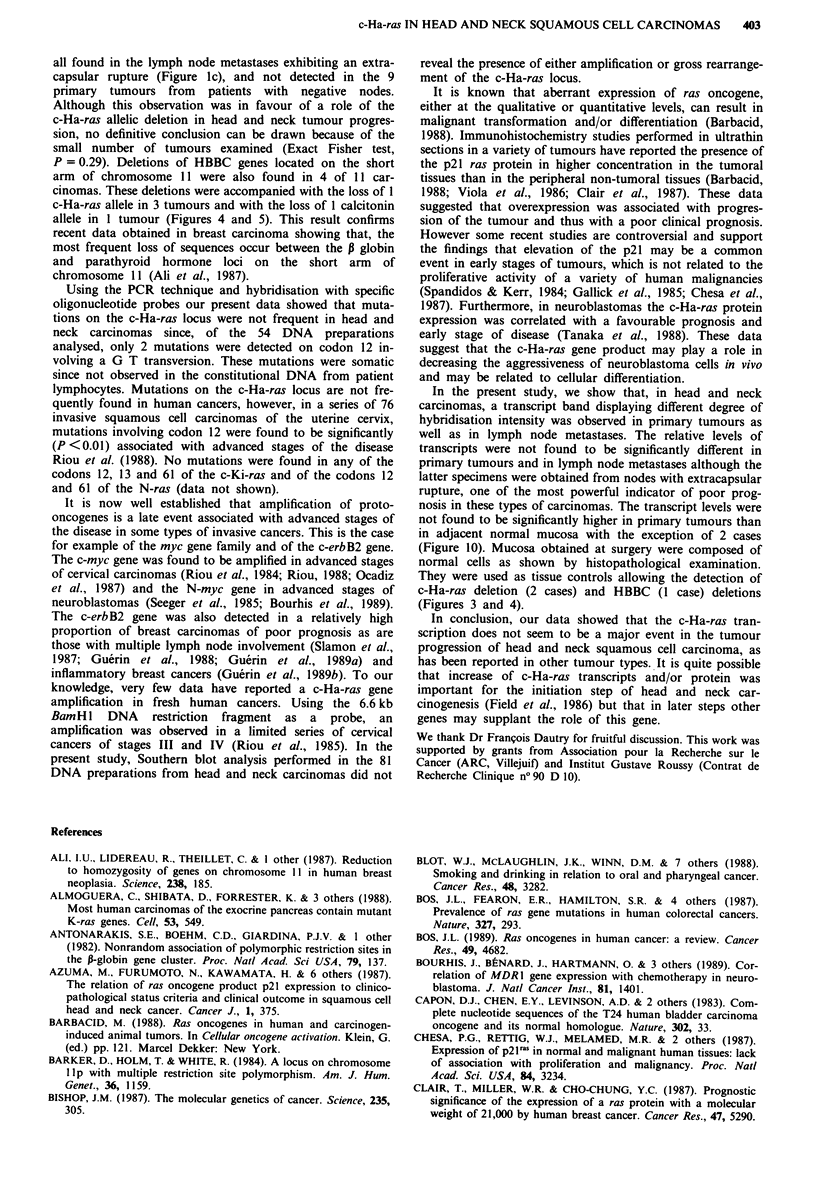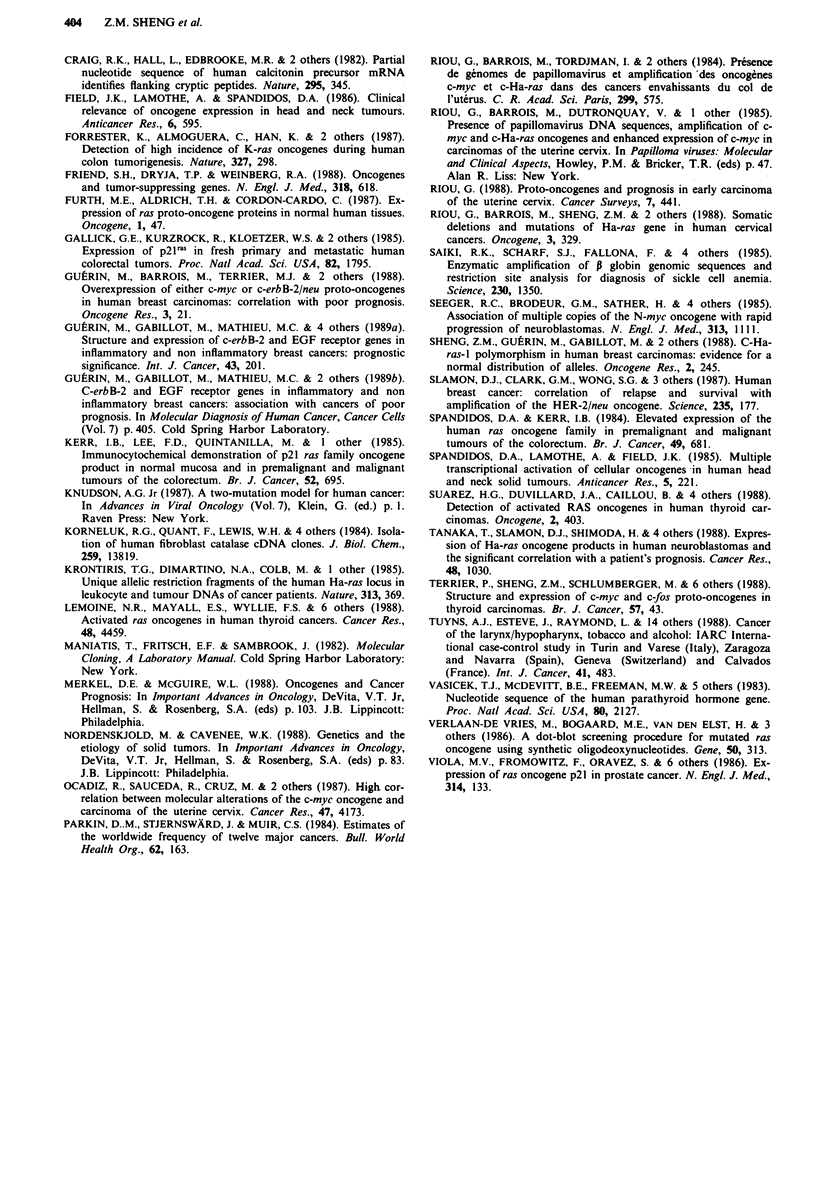# Analysis of the c-Ha-ras-1 gene for deletion, mutation, amplification and expression in lymph node metastases of human head and neck carcinomas.

**DOI:** 10.1038/bjc.1990.306

**Published:** 1990-09

**Authors:** Z. M. Sheng, M. Barrois, J. Klijanienko, C. Micheau, J. M. Richard, G. Riou

**Affiliations:** Laboratoire de Pharmacologie Clinique et Moléculaire, Institut Gustave Roussy, Villejuif, France.

## Abstract

**Images:**


Br. J. Cancer (1990), 62, 398-404 © Macmillan Press Ltd., 1990

### Analysis of the c-Ha-*ras*-1 gene for deletion, mutation, amplification and expression in lymph node metastases of human head and neck carcinomas

Z.M. Sheng, M. Barrois[1], J. Klijanienko[2], C. Micheau[2], J.M. Richard[3] & G. Riou[1]

[1]*Laboratoire de Pharmacologie Clinique et Moléculaire,* [2]*Service d'Histopathologie,* [3]*Département de Chirurgie Cervico-faciale, Institut Gustave Roussy, 94800 Villejuif, France.*

**Summary** The c-Ha-*ras* gene was analysed by Southern blot hybridisation in 67 specimens of lymph node metastases and in 25 specimens of primary tumours obtained from 85 untreated patients with head and neck squamous cell carcinoma. The loss of one c-Ha-*ras* allele was observed in 10/46 (22%) tumours from heterozygous patients for this locus. Different genes, located as the c-Ha-*ras* gene on the short arm of chromosome 11, were also found to be deleted suggesting that the deletion of other genes could play a role in aggressiveness of head and neck carcinomas. Using polymerase chain reaction, mutation at codon 12 was detected in only 2/54 (3.8%) tumours but no mutation involving codon 61 was found. Neither gene amplification nor gene rearrangement could be observed. Total RNA was prepared from 79 of these tumour specimens and analysed by Northern and slot blot hybridisation. A 1.2 kb c-Ha-*ras* transcript band was detected in all the RNA preparations. Relatively high c-Ha-*ras* transcript levels were found in 18% of lymph node metastases and in 21% of primary tumours, indicating no significant differences between these cancers. Moreover, the c-Ha-*ras* mRNA levels were not significantly greater in the primary tumours than in the normal mucosae in 10/12 cases for which both tissues were analysed. These data indicate that c-Ha-*ras* gene does not seem to be strongly involved in head and neck carcinomas at that advanced stage of the disease, as this was previously reported for earlier clinical stages.

It is now well established that genetic alterations are implicated in the biological deregulation of cancer cells and that the cellular oncogenes are involved in the cancer process (Bishop, 1987; Merkel & McGuire, 1988; Nordenskjold & Cavenee, 1988). Among those cellular oncogenes, the *ras* genes were thought to play an important role and many studies were initiated to detect alterations and aberrant expression of these genes. Somatic mutations, resulting in the substitution of a single base at particular positions in the gene locus were found to be responsible for oncogenic activity by transfection assay, in about 15% of cancers (Barbacid, 1988). Recently, using more sensitive methods, high rates of mutation affecting the c-Ki-*ras* gene were detected in DNA from pancreas and colon carcinomas (Bos *et al.*, 1987; Forrester *et al.*, 1987; Almoguera *et al.*, 1988; Bos, 1989). In colon tissues the c-Ki-*ras* mutations were even found in the precancerous lesions which were assumed to progress in invasive carcinomas. Mutations of the *ras* gene were also found in thyroid adenomas and carcinomas (Lemoine *et al.*, 1988; Suarez *et al.*, 1988). C-Ha-*ras* mutation at codon 12 was shown to be associated with cervical cancers of poor prognosis (Riou *et al.*, 1988). A c-Ha-*ras* restriction fragment length polymorphism (RFLP) (Capon *et al.*, 1983) linked to susceptibility of individuals to cancers was reported (Krontiris *et al.*, 1985) and deletions of the c-Ha-*ras* locus were observed in a variety of human cancers (Nordenskjold & Cavenee, 1988; Riou *et al.*, 1988; Sheng *et al.*, 1988). Other alterations of the *ras* gene consisting of amplification or rearrangement were more rarely found in human tumours (Barbacid, 1988). On the contrary, studies on the expression of *ras* genes indicated that high levels of *ras*-specific messenger RNA and *ras* p21 protein were associated with tumour progression in human cancers of different origins (Viola *et al.*, 1986; Clair *et al.*, 1987). However studies have shown the presence of high levels of *ras* transcripts or proteins in normal tissues (Furth *et al.*, 1987; Kerr *et al.*, 1985) while others have reported that high levels of these gene transcripts or protein were not necessarily related to tumour progression (Spandidos & Kerr, 1984; Gallick *et al.*, 1985; Chesa *et al.*, 1987).

In this study we have analysed the c-Ha-*ras*-1 for allelic deletion, mutation, amplification and expression in the same specimens of a large series of lymph node metastases from patients with head and neck squamous cell carcinomas. Our aim was to determine whether the c-Ha-*ras* proto-oncogene by alteration of its structure and/or overexpression plays also a role in the progression of these cancers known to be of poor prognosis.

Correspondence: G. Riou.
Received 2 April 1990; and in revised form 8 May 1990.

### Materials and methods

### *Carcinomas and control specimens*

Ninety-two specimens of head and neck squamous cell carcinoma of different locations were obtained by surgical excision from 85 untreated patients (Table I); 25 samples were primary tumours and 67 samples were lymph node metastases. We could obtain both primary tumours and lymph node metastases from 7 patients and primary tumours and normal mucosa from 14 other patients. In each case, normal mucosa was cut off distantly from the tumour. Histopathological examination was performed on all the tissue specimens (Figure 1a, b and c). Carcinomas were of squamous cell histological type. A large marjority of these carcinomas were well differentiated. Lymphocytes were obtained from 65 healthy donors and 15 patients. The lymphocytes were fractionated in a Ficoll-Hypaque gradient. All the tissue and cell samples were immediately stored in liquid nitrogen.

### *Isolation of DNA and RNA*

DNA and RNA were prepared from the same tissue samples corresponding to about 50–200 mg of fresh tissue. Frozen tissues were ground in liquid nitrogen and nucleic acids extracted by the guanidinium-thiocyanate method. DNA and RNA were fractionated by centrifugation in a CsCl gradient (Maniatis *et al.*, 1982; Terrier *et al.*, 1988). Nucleic acids were also prepared from EJ human cancer cell line. EJ cells originated from a bladder carcinoma with transitional cells exhibiting on the c-Ha-*ras* gene a point mutation at codon 12 involving a transversion G T.

### *Southern blots*

DNA preparations (5 or 10 μg) were incubated with H*pa*II-M*sp*I enzymes to analyse c-Ha-*ras* RFLP and allele loss and with *Sac*I enzyme to analyse the structure of the locus.

Southern blot hybridisations using the different probes were performed. Other genes located on the short arm of chromosome 11 were also studied for allelic loss. Different restriction enzymes and probes were used as noted in the legend of Figure 2.

*Northern blots*

Denatured total RNA samples (10 µg per well) fractionated on a 1.2% formaldehyde agarose gel and transferred to a Hybond C extra filter were analysed by Northern blot hybridisation as previously described (Maniatis *et al.*, 1982). The quality and the quantity of total RNA were tested by a preliminary electrophoresis in a non-denaturing 1.2% agarose mini-gel. The filters were exposed at −70°C to Kodak XAR 5 films for various periods of times.

**Figure 1** Histological sections. Normal mucosa **a**, and squamous cell carcinoma **b**, were obtained from larynx by surgery. Carcinoma was composed of typically well differentiated squamous cells. Lymph node metastatic tumour exhibiting extracapsular rupture **c**, arrow. (Hematoxylin-eosin stain. **a**, **b** × 136; **c**, × 21.25).

**Figure 2** Schematic representation of the 6.6 kb *Bam*H1 DNA fragment carrying the human c-Ha-*ras*1 gene obtained from EJ cells. The black boxes represent the 4 exons and the hatched box, the region of variable tandem repetition (VTR). The 2.5 kb *Sac*I-*Bam*H1 DNA fragment (probe 1) was used to detect RFLP and gene deletions, the 2.9 kb *Sac*I-*Sac*I DNA fragment (probe 2) encompassing the 4 exons was used to detect amplification and expression of the gene (**B**, *Bam*H1 and S, *Sac*I restriction sites).

*Slot blots*

Preparations of total RNA were applied to nitrocellulose filters (BA 85) using a slot blot apparatus (Schleicher & Schuell). Total RNA from EJ cell line was used to measure the levels of c-Ha-*ras* mRNA. Transcript level in this cell line was stable and arbitrarily considered as 1 unit. Transcript level was determined by densitometer scanning of the autoradiographic bands (Chromoscan 3, Joyce Loebl). To provide a control for the amount of RNA on the filters, the c-Ha-*ras* gene signal was removed and the same filters rehybridised with an actin probe.

*Detection of mutation by polymerase chain reaction (PCR)*

We have amplified sequences spanning 63–128 base pairs across codons 12, 13, and 61 of *ras* (Verlaan-de Vries *et al.*, 1986). Amplification was carried out as described by Saiki *et al.* (1985) using *Thermus aquaticus* DNA polymerase (Perkin-Elmer Cetus). All the oligonucleotides used in our laboratory were synthesised by the solid phase triester method. The 5′ ends of oligomers were labelled with T4 polynucleotide kinase (Maniatis *et al.*, 1982). Aliquots (2 µl) of the PCR mixture were spotted to a nylon filter (Gelman) and hybridised to a panel of 20 mers synthetic oligonucleotide probes. Hybridisation and washing of filters were performed in a 3 M tetramethylammonium chloride salt solution (Verlaan-de Vries *et al.*, 1986).

*Probes*

Two c-Ha-*ras* probes were used, the 2.5 kilobase pairs (kb) *Bam*H1-*Sac*I DNA fragment (probe 1) encompassing the Variable Tandem Repetitive (VTR) region (Capon *et al.*, 1983) and the 2.9 kb *Sac*I-*Sac*I DNA fragment encompassing the c-Ha-*ras* coding exons (probe 2). These probes were obtained from the 6.6 kb c-Ha-*ras*–1 gene of EJ cells. Probes used as markers on chromosome 11 were: D11S12 from clone pADJ762 (Barker *et al.*, 1984); HBBC (γG and γA of the β globin cluster) from clone JW151 (Antonarakis *et al.*, 1982); PTH (human parathyroid hormone) from clone pPTH 2.5 (Vasicek *et al.*, 1983); CALC (calcitonin) from clone phTB3 (Craig *et al.*, 1982); CAT (catalase) from clone pCAT41 (Korneluk *et al.*, 1984).

### Results

Characteristics of head and neck carcinomas are presented in Table I. A large majority of the carcinomas which were analysed, were of poor prognosis since patients had lymph node involvement with extracapsular rupture, one of the most powerful indicator of prognosis in these types of cancer (Figure 1c). RFLP was analysed in DNA preparations from carcinomas and from lymphocytes of healthy blood donors. A RFLP for *Bam*HI or *Msp*I (Capon *et al.*, 1983; Krontiris *et al.*, 1985) was found to occur in a region of variable tandem repetition (VTR) located at the 3′ end of the c-Ha-*ras* locus (Figure 2). After Southern blot hybridisation using the c-Ha-*ras*-1 VTR probe (probe 1, Figure 2), several classes of allelic restriction fragments were observed, some of them were frequently detected while others rarely found (Krontiris *et al.*, 1985). Single or 2 DNA fragments were revealed in cancer DNA preparations from homozygous and heterozygous patients respectively. Figure 3A shows representative patterns of hybridisation from tumour DNAs. Similar patterns were obtained from DNAs of unaffected population (data not shown). The size and the frequency of the different alleles are presented in Table II. The rare alleles occurred with similar combined frequencies in head and neck patients (7%) and in normal individuals (6.5%). As expected, identical c-Ha-*ras*-1 VTR alleles were detected in DNAs from tumours and lymphocytes or normal mucosae obtained from the same patients. Heterozygosity for this allele was found in 46 tumours and the loss of 1 allele was observed in the tumour samples from 10 (22%) of these heterozygous patients (Figure 3B).

**Table I** Characteristics of squamous cell head and neck carcinomas and tissues

| Site of the primary tumour | Total no. of patients | No. of node positive patients | No. of patients with at least one node with extracapsular rupture | No. of samples analysed[e] Primary tumour | Node metastasis | Normal mucosa[f] |
|---|---|---|---|---|---|---|
| Hypopharynx[a] | 22 | 22 | 20 | 6 | 19 | 4 |
| Oral cavity[b] | 21 | 21 | 20 | 5 | 18 | 1 |
| Larynx[c] | 18 | 14 | 14 | 5 | 14 | 5 |
| Oropharynx[c] | 14 | 13 | 12 | 4 | 11 | 3 |
| Sinus and hard palate | 5 | 1 | 1 | 4 | 1 | 1 |
| Unknown[d] | 5 | 5 | 5 | 1[g] | 4 | |
| Total | 85 | 76 | 72 | 25 | 67 | 14 |

[a,b,c]Both primary tumour and metastasis from 3[a], 2[b] and 1[c] patients were analysed. [d]Lymphadenopathy with unknown site of the primary tumour. [e]DNA from the 92 tumour samples were analysed whereas RNA from 79 of these samples were analysed. [f]Both normal mucosa and primary tumour from 14 patients were analysed. [g]The sample quoted with primary tumour was a tumoral mass of unknown origin.

**Figure 3** Southern blot analysis of c-Ha-*ras*-1 restriction fragment length polymorphism in head and neck squamous cell carcinomas and normal cells from patients (normal mucosa, N; lymphocytes, L). DNA samples (5 μg) were digested with *Hpa*II-*Msp*I, electrophoresed in 2% agarose gel and hybridised with the c-Ha-*ras*-1-VTR probe (probe 1 of Figure 2). DNA with similar-sized alleles were re-electrophoresed in the same gel in separated wells and in the same well. **A**, head and neck carcinomas. **B**, pairs L-Ca (carcinoma) and N-Ca from the same patients. Note the loss of 1 allele in $Ca_1$, $Ca_2$ and $Ca_3$. A faint hybridisation band to the deleted band probably due to the presence of normal cells in tumours was observed in $Ca_1$ and $Ca_3$.

**Table II** C-Ha-*ras* allele frequencies in head and neck carcinomas and normal lymphocytes

| Allele size (kb) | Allele frequencies (%) Lymphocytes of healthy donors (65)[a] | Carcinomas (71)[a] |
|---|---|---|
| Common alleles | | |
| 1.1 | 66.0 | 54.9 |
| 1.6 | 14.5 | 12.7 |
| 2.2 | 6.5 | 9.9 |
| 2.6 | 6.5 | 15.5 |
| Total | 93.5 | 93.0 |
| Rare alleles | | |
| 0.95 to 2.8 | 6.5 | 7.0 |

[a]In parentheses the number of samples analysed.

Several genes located in the vicinity of the c-Ha-*ras* locus on the short arm of chromosome 11 were also analysed to determine the extension of chromosome deletion. An allelic loss pattern after Southern blot hybridisation (Figure 4) and a schematic representation of the deleted genes (Figure 5) are presented. The allelic deletions for different loci on chromosome 11p were observed in 4 of the 11 head and neck carcinomas analysed. One HBBC allele belonging to the β globin cluster was lost in these 4 carcinomas together with 1 c-Ha-*ras* allele in 3 carcinomas and with 1 calcitonin allele in the other carcinoma.

The presence of mutation at positions 12 and 61 on the c-Ha-*ras* locus was investigated in 54 carcinoma specimens using the PCR technique and hybridisation with synthesised specific probes. A mutation affecting the codon 12 as in the EJ cell lines was observed in only 2 carcinomas and not detected in the lymphocyte specimens. The same transversion G T was observed in the 2 cases of mutated c-Ha-*ras* genes (Figure 6). No mutation was detected at position 61. The presence of mutation at positions 12, 13 and 61 on the Ki-*ras* gene and of mutation at positions 12 and 61 on the N-*ras* gene was also investigated in 28 carcinoma samples. No mutation could be evidenced (data not shown). DNA preparations, after digestion with *Sac*I, were analysed by Southern blot hybridisation with the probe 2 (Figure 2) encompassing the 4 c-Ha-*ras* exons. As expected a 2.9 kb DNA band was observed in all the DNA preparations. Neither gene amplification nor gross gene rearrangement were found in carcinomas (Figure 7).

The total RNA was prepared from 79 specimens of these carcinomas and from 12 specimens of normal mucosa. RNA preparations were analysed by Northern and slot blot hybridisation using the c-Ha-*ras*-1 gene probe 2 of Figure 2. As usually observed in human tissues, a 1.2 kb transcript band was detected in all RNA preparations. A representative Northern blot was shown in Figure 7. Blots were deshybridised then rehybridised with the actin probe which was assumed to be equally expressed in cancers. The levels of c-Ha-*ras* gene transcripts were determined by densitometric analysis of autoradiograms from slot blots and normalised against actin values (Figure 8). Relative levels were evaluated by comparison to c-Ha-*ras* transcript level obtained in EJ cells. Lymph node metastases and primary tumours were independently grouped according to their relative c-Ha-*ras* mRNA levels (Figure 9). The mean values (in arbitrary units ± standard deviation) were equal to 0.8 (± 0.9) in metastases and 0.9 (± 0.6) in primary tumours. The differences were not found to be significant (Student Fisher test $P = 0.4$). The c-Ha-*ras* mRNA levels found in individual primary tumours and normal mucosae node metastases are presented in Figure 10. In only 2 of 12 cases, the c-Ha-*ras* transcript level was found to be significantly higher in tumour than in normal mucosa. No significant difference was observed between primary tumours and lymph node metastases. As shown in Table III the level of c-Ha-*ras* RNA expression was not influenced by the state of c-Ha-*ras* gene in tumours (loss of allele, mutation, or presence of rare allele).

**Figure 4** Allelic deletions on chromosome 11p in head and neck carcinomas. Southern blot hybridisation, DNA from normal mucosa (N) and primary tumour (T) of a head and neck patient with a larynx location (Patient d Figure 5). Restriction enzymes used were *Hpa*II/*Msp*I for c-Ha-*ras* and D11S12 loci, *Hind*III for HBBC, *Pst*I for parathyroid hormone (PTH) locus and *Taq*I for calcitonin (CALC) and catalase (CAT) loci. Allelic deletions were observed for HBBC and CALC, the presence of a faint 8 kb hybridisation band was due to the presence of normal cells in these tumours. Only sizes in kb of DNA bands which could undergo an allele loss are given.

**Figure 5** Map of the common region of 11p deletion. Positions of 6 genes or markers from chromosome 11p are shown, a black box indicates that 1 parental allele was lost in the carcinoma, a hatched box indicates that both parental alleles were retained in the carcinoma, an open box indicates that the marker was not informative (the patient's normal mucosa was homozygous for the marker). An allele loss (apolipoprotein 1, Apo A1) on chromosome 11q is also shown.

**Figure 6** Dot hybridisation of *in vitro*-amplification DNA by PCR from head and neck carcinoma and EJ cells with oligomer probes corresponding to Gly (wild type) and Val of c-Ha-*ras* codon 12. Only 2 mutations (lanes 1A and 1C) affecting Ha-*ras* were observed in carcinomas and in EJ cells (lane 7B).

**Figure 7** Analysis of DNA and total RNA prepared from the same specimens of head and neck carcinoma for amplification and expression of the c-Ha-*ras* gene. EJ, cell line; Ca, carcinoma; N, normal mucosa. **A**, Southern blot analysis: DNA (5 µg per well) was incubated with *Sac*I restriction endonculease. **B**, Northern blot analysis of total RNA (10 µg per well). Filters were sequentially hybridised with the c-Ha-*ras* probe then actin probe; kb, kilobase pair, c-Ha-*ras*-1 probe 2 (Figure 2).

**Figure 8** Analysis of c-Ha-*ras* transcripts by slot blot hybridisation (5 µg of total RNA in each slot). Filters were sequentially hybridised with the c-Ha-*ras*-1 probe (probe 2 of Figure 2) and the actin probe. Panels A, B, C lanes 1 to 10, RNA from head and neck carcinoma except in B lane 10, RNA from EJ cell line. Panel D, lanes 1–2, 3–4 and 5–6, carcinoma lymph-node metastasis and lanes 7–8 and 9–10, primary tumour-normal mucosa pairs from the same patients.

**Figure 9** Expression of c-Ha-*ras* proto-oncogene in 60 specimens of lymph node metastases (M) and 19 specimens of primary tumours (T) from head and neck carcinomas of various locations (see Table I) (at the top of each column, the number of carcinomas per group). The level of c-Ha-*ras* transcripts in each carcinoma was evaluated relatively to the level found in EJ cells (1 unit). The values were measured by densitometer analysis (Chromoscan 3, Joyce Loebl) of slot blot autoradiograms (see Figure 8). Controls for amount of total RNA in each slot were provided by actin signal, considering that actin mRNA levels did not vary in carcinomas.

**Figure 10** Relative levels of c-Ha-*ras* expression in pairs primary tumour (T, black stripe)-normal mucosa (N, white stripe) and pairs primary tumour (black stripe)-lymph node metastasis (M, cross-hatched stripe). For quantitation see legend of Figure 8 and Materials and methods.

**Table III** Expression of the c-Ha-*ras* gene according to its structural characteristics

| *Characteristics of c-Ha-ras in individual carcinomas* | *Relative level of c-Ha-ras mRNA* |
|---|---|
| Allelic deletion | |
| $O_{138}$[a] | 0.39 |
| $O_2$ | 0.42 |
| $O_{42}$ | 0.62 |
| $O_{55}$ | 0.69 |
| $O_8$ | 0.85 |
| $O_{33}$ | 2 |
| $O_{140}$ | 3.4 |
| Mutation at codon 12 | |
| $O_{52}$ | 0.38 |
| $O_{50}$ | 0.54 |
| Rares alleles | |
| $O_{22}$ | 0.4 |
| $O_{37}$ | 0.69 |
| $O_4$ | 0.92 |
| $O_{54}$ | 1.2 |
| $O_{60}$ | 1.3 |

[a]Patient no.

### Discussion

Head and neck cancers are among the commonest cancers world-wide (Parkin *et al.*, 1984) and the large geographical variations in incidence have suggested the importance of environmental risk factors (Blot *et al.*, 1988; Tuyns *et al.*, 1988). Epidemiological studies have shown that these cancers, if we except carcinomas of the nasopharynx for which Epstein-Barr virus is the main aetiological factor, were strongly associated with a high intake of alcohol and tobacco smoking. The majority of carcinomas of this study developed in patients with poor prognosis since specimens were obtained from lymph node metastases exhibiting extracapsular rupture (Table I, Figure 1c).

Studies have reported that an aberrant expression of the c-Ha-*ras* gene was associated with tumoral progression (Viola *et al.*, 1986; Clair *et al.*, 1987; Spandidos *et al.*, 1985; Azuma *et al.*, 1987). If the c-Ha-*ras* gene was involved in head and neck tumour progression, we should expect to find c-Ha-*ras* gene alteration or overexpression with a high frequency in these very aggressive cancers. We have analysed the c-Ha-*ras*-1 RFLP for *Hpa*II-*Msp*I enzymes in these carcinomas comparatively to a control population of healthy blood donors and found no significant differences in the alleles frequencies. Consequently, the presence of variant alleles at the c-Ha-*ras*-1 locus does not seem to be an informative marker for the genetic predisposition to head and neck carcinomas, as has been suggested in cancers of other origins (Krontiris *et al.*, 1985).

Recent studies have shown that allelic deletions could signal the presence of a tumour suppressor gene within the affected regions of the chromosome. When both allelic copies of such a gene are inactivated, suppression may be relieved resulting in an abnormal proliferation. This process was particularly well documented for retinoblastoma and Wilm's tumour, 2 childhood tumours (Knudson, 1987; Friend *et al.*, 1988). The tumour suppressor gene concept was extended to other common adult malignancies but the numerous studies undertaken until now have still not clearly shown the involvement of such suppressor genes. Rather, these studies have reported chromosomal deletions affecting particular loci in certain carcinomas, suggesting a prevalent role of these alterations in the development of malignancies. Deletions on the short arm of chromosome 11 were considered as being a common and important event in the development of many cancers and sometimes in tumour progression. Of the 46 head and neck carcinomas whose patients were constitutionally heterozygous for the c-Ha-*ras* locus, the loss of 1 allele was detected in 10 (22%) specimens. The deletions were all found in the lymph node metastases exhibiting an extracapsular rupture (Figure 1c), and not detected in the 9 primary tumours from patients with negative nodes. Although this observation was in favour of a role of the c-Ha-*ras* allelic deletion in head and neck tumour progression, no definitive conclusion can be drawn because of the small number of tumours examined (Exact Fisher test, $P = 0.29$). Deletions of HBBC genes located on the short arm of chromosome 11 were also found in 4 of 11 carcinomas. These deletions were accompanied with the loss of 1 c-Ha-*ras* allele in 3 tumours and with the loss of 1 calcitonin allele in 1 tumour (Figures 4 and 5). This result confirms recent data obtained in breast carcinoma showing that, the most frequent loss of sequences occur between the β globin and parathyroid hormone loci on the short arm of chromosome 11 (Ali *et al.*, 1987).

Using the PCR technique and hybridisation with specific oligonucleotide probes our present data showed that mutations on the c-Ha-*ras* locus were not frequent in head and neck carcinomas since, of the 54 DNA preparations analysed, only 2 mutations were detected on codon 12 involving a G T transversion. These mutations were somatic since not observed in the constitutional DNA from patient lymphocytes. Mutations on the c-Ha-*ras* locus are not frequently found in human cancers, however, in a series of 76 invasive squamous cell carcinomas of the uterine cervix, mutations involving codon 12 were found to be significantly ($P < 0.01$) associated with advanced stages of the disease Riou *et al.* (1988). No mutations were found in any of the codons 12, 13 and 61 of the c-Ki-*ras* and of the codons 12 and 61 of the N-*ras* (data not shown).

It is now well established that amplification of proto-oncogenes is a late event associated with advanced stages of the disease in some types of invasive cancers. This is the case for example of the *myc* gene family and of the c-*erb*B2 gene. The c-*myc* gene was found to be amplified in advanced stages of cervical carcinomas (Riou *et al.*, 1984; Riou, 1988; Ocadiz *et al.*, 1987) and the N-*myc* gene in advanced stages of neuroblastomas (Seeger *et al.*, 1985; Bourhis *et al.*, 1989). The c-*erb*B2 gene was also detected in a relatively high proportion of breast carcinomas of poor prognosis as are those with multiple lymph node involvement (Slamon *et al.*, 1987; Guérin *et al.*, 1988; Guérin *et al.*, 1989*a*) and inflammatory breast cancers (Guérin *et al.*, 1989*b*). To our knowledge, very few data have reported a c-Ha-*ras* gene amplification in fresh human cancers. Using the 6.6 kb *Bam*H1 DNA restriction fragment as a probe, an amplification was observed in a limited series of cervical cancers of stages III and IV (Riou *et al.*, 1985). In the present study, Southern blot analysis performed in the 81 DNA preparations from head and neck carcinomas did not reveal the presence of either amplification or gross rearrangement of the c-Ha-*ras* locus.

It is known that aberrant expression of *ras* oncogene, either at the qualitative or quantitative levels, can result in malignant transformation and/or differentiation (Barbacid, 1988). Immunohistochemistry studies performed in ultrathin sections in a variety of tumours have reported the presence of the p21 *ras* protein in higher concentration in the tumoral tissues than in the peripheral non-tumoral tissues (Barbacid, 1988; Viola *et al.*, 1986; Clair *et al.*, 1987). These data suggested that overexpression was associated with progression of the tumour and thus with a poor clinical prognosis. However some recent studies are controversial and support the findings that elevation of the p21 may be a common event in early stages of tumours, which is not related to the proliferative activity of a variety of human malignancies (Spandidos & Kerr, 1984; Gallick *et al.*, 1985; Chesa *et al.*, 1987). Furthermore, in neuroblastomas the c-Ha-*ras* protein expression was correlated with a favourable prognosis and early stage of disease (Tanaka *et al.*, 1988). These data suggest that the c-Ha-*ras* gene product may play a role in decreasing the aggressiveness of neuroblastoma cells *in vivo* and may be related to cellular differentiation.

In the present study, we show that, in head and neck carcinomas, a transcript band displaying different degree of hybridisation intensity was observed in primary tumours as well as in lymph node metastases. The relative levels of transcripts were not found to be significantly different in primary tumours and in lymph node metastases although the latter specimens were obtained from nodes with extracapsular rupture, one of the most powerful indicator of poor prognosis in these types of carcinomas. The transcript levels were not found to be significantly higher in primary tumours than in adjacent normal mucosa with the exception of 2 cases (Figure 10). Mucosa obtained at surgery were composed of normal cells as shown by histopathological examination. They were used as tissue controls allowing the detection of c-Ha-*ras* deletion (2 cases) and HBBC (1 case) deletions (Figures 3 and 4).

In conclusion, our data showed that the c-Ha-*ras* transcription does not seem to be a major event in the tumour progression of head and neck squamous cell carcinoma, as has been reported in other tumour types. It is quite possible that increase of c-Ha-*ras* transcripts and/or protein was important for the initiation step of head and neck carcinogenesis (Field *et al.*, 1986) but that in later steps other genes may supplant the role of this gene.

We thank Dr François Dautry for fruitful discussion. This work was supported by grants from Association pour la Recherche sur le Cancer (ARC, Villejuif) and Institut Gustave Roussy (Contrat de Recherche Clinique n° 90 D 10).

### References

ALI, I.U., LIDEREAU, R., THEILLET, C. & 1 other (1987). Reduction to homozygosity of genes on chromosome 11 in human breast neoplasia. *Science*, **238**, 185.

ALMOGUERA, C., SHIBATA, D., FORRESTER, K. & 3 others (1988). Most human carcinomas of the exocrine pancreas contain mutant K-*ras* genes. *Cell*, **53**, 549.

ANTONARAKIS, S.E., BOEHM, C.D., GIARDINA, P.J.V. & 1 other (1982). Nonrandom association of polymorphic restriction sites in the β-globin gene cluster. *Proc. Natl Acad. Sci USA*, **79**, 137.

AZUMA, M., FURUMOTO, N., KAWAMATA, H. & 6 others (1987). The relation of *ras* oncogene product p21 expression to clinicopathological status criteria and clinical outcome in squamous cell head and neck cancer. *Cancer J.*, **1**, 375.

BARBACID, M. (1988). *Ras* oncogenes in human and carcinogen-induced animal tumors. In *Cellular oncogene activation*. Klein, G. (ed.) pp. 121. Marcel Dekker: New York.

BARKER, D., HOLM, T. & WHITE, R. (1984). A locus on chromosome 11p with multiple restriction site polymorphism. *Am. J. Hum. Genet.*, **36**, 1159.

BISHOP, J.M. (1987). The molecular genetics of cancer. *Science*, **235**, 305.

BLOT, W.J., McLAUGHLIN, J.K., WINN, D.M. & 7 others (1988). Smoking and drinking in relation to oral and pharyngeal cancer. *Cancer Res.*, **48**, 3282.

BOS, J.L., FEARON, E.R., HAMILTON, S.R. & 4 others (1987). Prevalence of *ras* gene mutations in human colorectal cancers. *Nature*, **327**, 293.

BOS, J.L. (1989). *Ras* oncogenes in human cancer: a review. *Cancer Res.*, **49**, 4682.

BOURHIS, J., BÉNARD, J., HARTMANN, O. & 3 others (1989). Correlation of *MDR*1 gene expression with chemotherapy in neuroblastoma. *J. Natl Cancer Inst.*, **81**, 1401.

CAPON, D.J., CHEN, E.Y., LEVINSON, A.D. & 2 others (1983). Complete nucleotide sequences of the T24 human bladder carcinoma oncogene and its normal homologue. *Nature*, **302**, 33.

CHESA, P.G., RETTIG, W.J., MELAMED, M.R. & 2 others (1987). Expression of $p21^{ras}$ in normal and malignant human tissues: lack of association with proliferation and malignancy. *Proc. Natl Acad. Sci. USA*, **84**, 3234.

CLAIR, T., MILLER, W.R. & CHO-CHUNG, Y.C. (1987). Prognostic significance of the expression of a *ras* protein with a molecular weight of 21,000 by human breast cancer. *Cancer Res.*, **47**, 5290.

CRAIG, R.K., HALL, L., EDBROOKE, M.R. & 2 others (1982). Partial nucleotide sequence of human calcitonin precursor mRNA identifies flanking cryptic peptides. *Nature*, **295**, 345.

FIELD, J.K., LAMOTHE, A. & SPANDIDOS, D.A. (1986). Clinical relevance of oncogene expression in head and neck tumours. *Anticancer Res.*, **6**, 595.

FORRESTER, K., ALMOGUERA, C., HAN, K. & 2 others (1987). Detection of high incidence of K-*ras* oncogenes during human colon tumorigenesis. *Nature*, **327**, 298.

FRIEND, S.H., DRYJA, T.P. & WEINBERG, R.A. (1988). Oncogenes and tumor-suppressing genes. *N. Engl. J. Med.*, **318**, 618.

FURTH, M.E., ALDRICH, T.H. & CORDON-CARDO, C. (1987). Expression of *ras* proto-oncogene proteins in normal human tissues. *Oncogene*, **1**, 47.

GALLICK, G.E., KURZROCK, R., KLOETZER, W.S. & 2 others (1985). Expression of p21$^{ras}$ in fresh primary and metastatic human colorectal tumors. *Proc. Natl Acad. Sci. USA*, **82**, 1795.

GUÉRIN, M., BARROIS, M., TERRIER, M.J. & 2 others (1988). Overexpression of either c-*myc* or c-*erb*B-2/*neu* proto-oncogenes in human breast carcinomas: correlation with poor prognosis. *Oncogene Res.*, **3**, 21.

GUÉRIN, M., GABILLOT, M., MATHIEU, M.C. & 4 others (1989*a*). Structure and expression of c-*erb*B-2 and EGF receptor genes in inflammatory and non inflammatory breast cancers: prognostic significance. *Int. J. Cancer*, **43**, 201.

GUÉRIN, M., GABILLOT, M., MATHIEU, M.C. & 2 others (1989*b*). C-*erb*B-2 and EGF receptor genes in inflammatory and non inflammatory breast cancers: association with cancers of poor prognosis. In *Molecular Diagnosis of Human Cancer, Cancer Cells* (Vol. 7) p. 405. Cold Spring Harbor Laboratory.

KERR, I.B., LEE, F.D., QUINTANILLA, M. & 1 other (1985). Immunocytochemical demonstration of p21 *ras* family oncogene product in normal mucosa and in premalignant and malignant tumours of the colorectum. *Br. J. Cancer*, **52**, 695.

KNUDSON, A.G. Jr (1987). A two-mutation model for human cancer: In *Advances in Viral Oncology* (Vol. 7), Klein, G. (ed.) p. 1. Raven Press: New York.

KORNELUK, R.G., QUANT, F., LEWIS, W.H. & 4 others (1984). Isolation of human fibroblast catalase cDNA clones. *J. Biol. Chem.*, **259**, 13819.

KRONTIRIS, T.G., DIMARTINO, N.A., COLB, M. & 1 other (1985). Unique allelic restriction fragments of the human Ha-*ras* locus in leukocyte and tumour DNAs of cancer patients. *Nature*, **313**, 369.

LEMOINE, N.R., MAYALL, E.S., WYLLIE, F.S. & 6 others (1988). Activated *ras* oncogenes in human thyroid cancers. *Cancer Res.*, **48**, 4459.

MANIATIS, T., FRITSCH, E.F. & SAMBROOK, J. (1982). *Molecular Cloning, A Laboratory Manual.* Cold Spring Harbor Laboratory: New York.

MERKEL, D.E. & MCGUIRE, W.L. (1988). Oncogenes and Cancer Prognosis: In *Important Advances in Oncology*, DeVita, V.T. Jr, Hellman, S. & Rosenberg, S.A. (eds) p. 103. J.B. Lippincott: Philadelphia.

NORDENSKJOLD, M. & CAVENEE, W.K. (1988). Genetics and the etiology of solid tumors. In *Important Advances in Oncology*, DeVita, V.T. Jr, Hellman, S. & Rosenberg, S.A. (eds) p. 83. J.B. Lippincott: Philadelphia.

OCADIZ, R., SAUCEDA, R., CRUZ, M. & 2 others (1987). High correlation between molecular alterations of the c-*myc* oncogene and carcinoma of the uterine cervix. *Cancer Res.*, **47**, 4173.

PARKIN, D.M., STJERNSWÄRD, J. & MUIR, C.S. (1984). Estimates of the worldwide frequency of twelve major cancers. *Bull. World Health Org.*, **62**, 163.

RIOU, G., BARROIS, M., TORDJMAN, I. & 2 others (1984). Présence de génomes de papillomavirus et amplification des oncogènes c-*myc* et c-Ha-*ras* dans des cancers envahissants du col de l'utérus. *C. R. Acad. Sci. Paris*, **299**, 575.

RIOU, G., BARROIS, M., DUTRONQUAY, V. & 1 other (1985). Presence of papillomavirus DNA sequences, amplification of c-*myc* and c-Ha-*ras* oncogenes and enhanced expression of c-*myc* in carcinomas of the uterine cervix. In *Papilloma viruses: Molecular and Clinical Aspects*, Howley, P.M. & Bricker, T.R. (eds) p. 47. Alan R. Liss: New York.

RIOU, G. (1988). Proto-oncogenes and prognosis in early carcinoma of the uterine cervix. *Cancer Surveys*, **7**, 441.

RIOU, G., BARROIS, M., SHENG, Z.M. & 2 others (1988). Somatic deletions and mutations of Ha-*ras* gene in human cervical cancers. *Oncogene*, **3**, 329.

SAIKI, R.K., SCHARF, S.J., FALLONA, F. & 4 others (1985). Enzymatic amplification of $\beta$ globin genomic sequences and restriction site analysis for diagnosis of sickle cell anemia. *Science*, **230**, 1350.

SEEGER, R.C., BRODEUR, G.M., SATHER, H. & 4 others (1985). Association of multiple copies of the N-*myc* oncogene with rapid progression of neuroblastomas. *N. Engl. J. Med.*, **313**, 1111.

SHENG, Z.M., GUÉRIN, M., GABILLOT, M. & 2 others (1988). C-Ha-*ras*-1 polymorphism in human breast carcinomas: evidence for a normal distribution of alleles. *Oncogene Res.*, **2**, 245.

SLAMON, D.J., CLARK, G.M., WONG, S.G. & 3 others (1987). Human breast cancer: correlation of relapse and survival with amplification of the HER-2/*neu* oncogene. *Science*, **235**, 177.

SPANDIDOS, D.A. & KERR, I.B. (1984). Elevated expression of the human *ras* oncogene family in premalignant and malignant tumours of the colorectum. *Br. J. Cancer*, **49**, 681.

SPANDIDOS, D.A., LAMOTHE, A. & FIELD, J.K. (1985). Multiple transcriptional activation of cellular oncogenes in human head and neck solid tumours. *Anticancer Res.*, **5**, 221.

SUAREZ, H.G., DUVILLARD, J.A., CAILLOU, B. & 4 others (1988). Detection of activated RAS oncogenes in human thyroid carcinomas. *Oncogene*, **2**, 403.

TANAKA, T., SLAMON, D.J., SHIMODA, H. & 4 others (1988). Expression of Ha-*ras* oncogene products in human neuroblastomas and the significant correlation with a patient's prognosis. *Cancer Res.*, **48**, 1030.

TERRIER, P., SHENG, Z.M., SCHLUMBERGER, M. & 6 others (1988). Structure and expression of c-*myc* and c-*fos* proto-oncogenes in thyroid carcinomas. *Br. J. Cancer*, **57**, 43.

TUYNS, A.J., ESTEVE, J., RAYMOND, L. & 14 others (1988). Cancer of the larynx/hypopharynx, tobacco and alcohol: IARC International case-control study in Turin and Varese (Italy), Zaragoza and Navarra (Spain), Geneva (Switzerland) and Calvados (France). *Int. J. Cancer*, **41**, 483.

VASICEK, T.J., MCDEVITT, B.E., FREEMAN, M.W. & 5 others (1983). Nucleotide sequence of the human parathyroid hormone gene. *Proc. Natl Acad. Sci. USA*, **80**, 2127.

VERLAAN-DE VRIES, M., BOGAARD, M.E., VAN DEN ELST, H. & 3 others (1986). A dot-blot screening procedure for mutated *ras* oncogene using synthetic oligodeoxynucleotides. *Gene*, **50**, 313.

VIOLA, M.V., FROMOWITZ, F., ORAVEZ, S. & 6 others (1986). Expression of *ras* oncogene p21 in prostate cancer. *N. Engl. J. Med.*, **314**, 133.